# Biocontrol of Carrot Disease-Causing Pathogens Using Essential Oils

**DOI:** 10.3390/plants10112231

**Published:** 2021-10-20

**Authors:** Simona Chrapačienė, Neringa Rasiukevičiūtė, Alma Valiuškaitė

**Affiliations:** Laboratory of Plant Protection, Lithuanian Research Centre for Agriculture and Forestry, Institute of Horticulture, Babtai, LT-54333 Kaunas, Lithuania; neringa.rasiukeviciute@lammc.lt (N.R.); alma.valiuskaite@lammc.lt (A.V.)

**Keywords:** *Alternaria* spp., oregano, essential oil, antifungal activity, plant protection

## Abstract

Diseases caused by fungal pathogens such as *Alternaria* spp. damage the commercial appearance of carrots or cause foliage diseases, resulting in significant yield losses each year and are a source of pre- and postharvest rots. European commission encourages the reduction of chemical pesticides. Therefore, the potential of essential oils for alternative plant protection is increasingly discussed. Furthermore, essential oils naturally produced by aromatic plants are rich in secondary metabolites, which possess several biological activities, and their use could be a significant step in environmentally friendly food production. This study aimed to evaluate the *Origanum vulgare* subsp. *vulgare* and *Origanum vulgare* subsp. *hirtum* essential oils efficacy on *Alternaria* spp. growth inhibition. A Clevenger-type apparatus was used to extract the essential oils from the fresh material. The *Alternaria* spp. radial colony growth was evaluated under essential oils concentrations from 200 to 600 µL L^−1^. Each essential oil separately was mixed with a PDA medium and *Alternaria* spp. disk placed in the center of the Petri dishes. Plates were incubated at 25 °C in the dark and evaluated 1, 2, 3, and 7 days after inoculation. The results revealed little difference between the essential oils, and the most effective concentration was 600 µL L^−1^ of *O. vulgare* subsp. *vulgare* essential oil and 400 µL L^−1^ of *O. vulgare* subsp. *hirtum*. Our findings can help to control carrot disease-causing pathogens *Alternaria* spp., but further research is needed.

## 1. Introduction

Essential components of global agriculture systems like agrochemicals and pesticides created a vital increase in crop yields and food production over the last century [1]. Nevertheless, worldwide studies have documented the pollution and consequence of agrochemical residues in soils, terrestrial and marine ecosystems [2,3,4,5]. In addition, several reports have declared that the increased number of pesticide-resistant strains of microorganisms, which demands the use of more concentrated chemicals, increases the risk of high-level toxic residues in fresh produce [6,7]. According to the Farm to Fork Strategy of the European Green Deal, there is an urgent need to reduce dependency on pesticides and antimicrobials, reduce excess fertilization, increase organic farming, and reverse biodiversity loss. The risk of using pesticides already decreased by 20% in the past five years. A further European Commission plan is to ensure the reduction of the overall use and risk of chemical pesticides by 50% by 2030 [8]. However, the rigorous regulations concerning the use and disposal of synthetic chemicals dramatically limit the possibility of perceiving future control strategies based only on pesticides. While chemical control is increasingly questioned because of its putative impact on the environment and human health, implementing alternative crop disease management solutions is imperative.

Carrot (*Daucus carota* L.) from the *Apiaceae* family is one of the popular root vegetables grown worldwide, rich in dietary carotenoids [9,10]. Carrot is biennial and produces a rosette of leaves on a compressed stem and a fleshy edible storage root in the first year of growth. This vegetable is also valued for its long shelf life at low temperatures. However, there are numerous carrot diseases responsible for significant yield losses [11,12]. Among them, *Alternaria* species are considered the most destructive and economically meaningful pathogens [13]. These necrotrophic fungi cause carrot Alternaria leaf blight and black rot, leading to pre- and postharvest damage. These diseases reduce carrots’ nutritional value, shelf life, and their resistance to diseases and destroy their aesthetic appearance. In the past, these infections did not cause significant losses to carrot growers. Still, over the past two decades, the harmfulness of pathogens of the genus *Alternaria* has increased dramatically worldwide. Depending on environmental conditions, cultivation technology, variety resistance, and other factors, yield losses can reach 40–60% [13,14].

Moreover, commercial carrot crops with weak and unhealthy foliage suffer the most because self-propelled multi-row mechanical harvesters are used with the grab belt system that catches the ground parts of the carrots and lifts them. Such harvesting requires robust, healthy foliage; otherwise, damaged leaves detach from the root, and carrots remain in the soil [14].

*Alternaria* fungi can produce more than 70 toxins, the most commonly studied natural contaminants in food are alternariol, alternariol monomethyl ether, and tentoxin, which play essential roles in fungal pathogenicity and food safety since some are toxic to plants and living organisms [14,15,16].

A wide diversity of biological control agents appear as safe and eco-friendly alternatives to pesticides to manage plant pathogens, while some even stimulate plant growth and improve soil structure at the same time [12,13]. For example, essential oils extracted from the leaves, stems, bark, and various parts of the plants contain complex mixtures of secondary metabolites, which are biologically active, endowed with antioxidant, allelopathic, bioregulatory, and antimicrobial properties. Other advantages of plant-based pesticides are their wide acceptance by consumers and potential multi-purpose uses [17,18,19,20].

*Origanum vulgare* L. belongs to the *Lamiaceae* family and is widely used as a spice and medicine. Furthermore, the potential of oregano as a protective agent in chronic-degenerative and infectious diseases was documented because of the anticancer, hepatoprotective, anti-inflammatory, antioxidant, and antimicrobial activities [21]. Therefore, it is believed that plant-derived extraction products of *O. vulgare* could be used in plant protection and prevent many pathogens’ growth and development. Furthermore, as this volatile oil is natural, non-phytotoxic, and biodegradable, it can overcome problems caused by chemical pesticides.

Reports on *Origanum vulgare* subsp. *vulgare* and *Origanum vulgare* subsp. *hirtum* efficacy against plant pathogens can be found in the literature [21,22,23,24,25,26]. It was previously found that extracts obtained from both plants effectively suppressed *Aspergillus niger*, *Aspergillus ochraceus*, and *Fusarium proliferatum* [24]. Another study [23] also reported a good antifungal activity against *Salmonella enteritidis*. In addition, the essential oils of both species exhibited moderate antibacterial and antifungal activities against *Sarcina lutea* and *Candida albicans* [25]. However, there is a lack of research on the effects of *O. vulgare* subsp. *vulgare* and *O. vulgare* subsp. *hirtum* essential oils on fungi of the genus *Alternaria*. Therefore, the current study aimed to evaluate the *O. vulgare* subsp. *vulgare* and *O. vulgare* subsp. *hirtum* essential oils efficacy on *Alternaria* spp. growth inhibition.

## 2. Results

**Figure 1:**
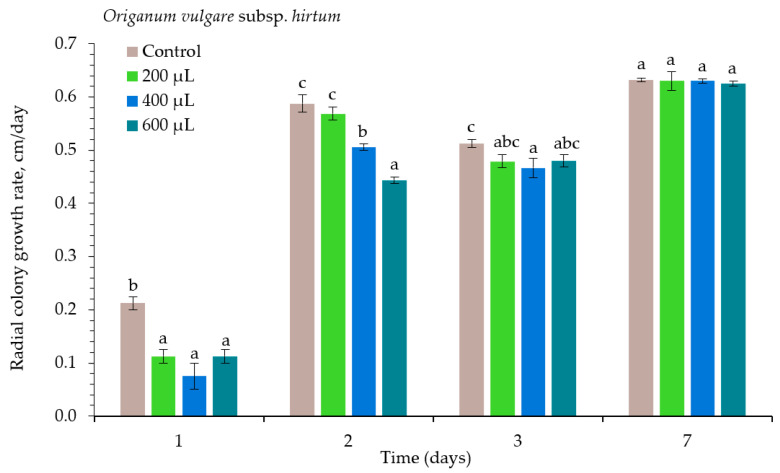
The radial colony growth rate of *Alternaria* spp. under the influence of *Origanum vulgare* subsp. *hirtum* essential oil. The same letter indicates no significant differences between treatments (*p* < 0.05).

The radial colony growth rate of the pathogens *Alternaria* spp. during the experimental period under the different concentrations of *Origanum vulgare* subsp. *hirtum* (OVH) essential oil (EO) is presented in Figure 1. The OVH EO at all tested concentrations suppressed the pathogen’s growth compared with the control at the beginning of the experiment. The lowest radial colony growth rate was at 400 µL L^−1^ 1 day after inoculation (DAI). The most considerable increase in colony growth was after 2 days. The concentration of 600 µL L^−1^ showed the most robust antifungal activity against *Alternaria* spp. At 200 µL L^−1^, the growth rate was very similar to the treatment with no EO. On the third assessment day, the EO effect on this pathogen was reduced, and mycelial growth was best controlled with a concentration of 400 µL L^−1^. This EO did not demonstrate significant suppression at 200, 400, and 600 µL L^−1^ at 7 DAI; the radial colony growth rates were comparable.

**Figure 2:**
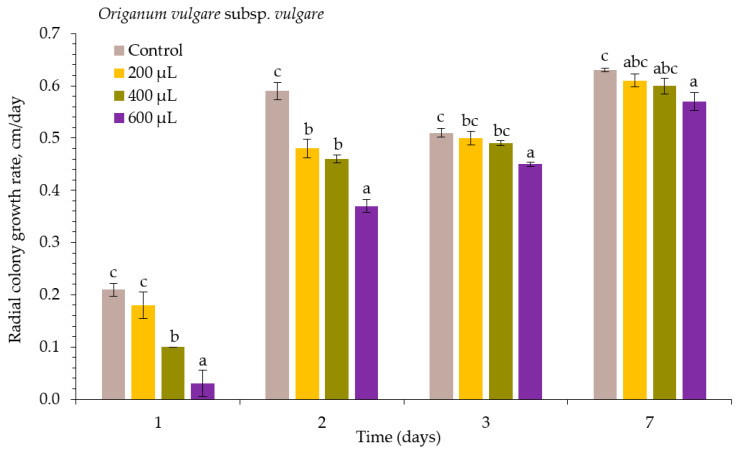
The radial colony growth rate of *Alternaria* spp. under the influence of *Origanum vulgare* subsp. *vulgare* essential oil. The same letter indicates no significant differences between treatments (*p* < 0.05).

The antifungal effect of different concentrations of *O. vulgare* subsp. *vulgare* (OVV) EO on *Alternaria* spp. radial colony growth rate is presented in Figure 2. Throughout the experiment, suppression of mycelial growth increased with EO concentration. Treatment with 600 µL L^−1^ of OVV EO showed the best antifungal activity against this pathogen and significantly differed from the control at 1, 2, 3, and 7 DAI. The radial colony growth rates at 200 and 400 µL L^−1^ were similar at 2, 3, and 7 DAI, as no significant differences were observed.

Mycelial growth inhibition was determined according to the radial colony growth rates for different treatments (Table 1). The results demonstrate that OVH EO showed low antifungal activity on *Alternaria* species compared to the control at 3 and 7 DAI. By contrast, the application of OVV EO showed higher inhibition compared to the control.

The assay revealed that the investigated *O. vulgare* subsp. *hirtum* EO and *O. vulgare* subsp. *vulgare* EO concentrations of 200–600 μL L^−1^ have unequal potential to suppress colony growth of *Alternaria* spp. Higher OVV oil concentrations, like 600 μL L^−1^, significantly inhibited fungal growth. However, OVH oil suppression was inefficient: the best and consistent result was shown with 400 μL L^−1^.

## 3. Discussion

The importance of reducing the environmental pollution in the agricultural and food sectors requires searching for alternative plant protection products. This study evaluated OVV and OVH EO efficiency on *Alternaria* spp. Many attempts have been made to discover alternative ways of controlling *Alternaria* spp. and reducing the use of fungicides [11,13,17,20,21]. Essential oils and plant extracts are two of the most promising groups of natural compounds for the development of safer fungicidal agents. There are significant results with *Thymus vulgaris* L., *Lavandula angustifolia* Mill., *Allium sativum* L., *Zingiber officinale* Roscoe, and other EO efficacy to control the growth of these pathogens [17,20].

The general antifungal activity of oregano extraction products was studied on microscopic fungi like *Ryzopus stolonifer*, *Penicillium digitatum*, *Botrytis cinerea*, *A. ochraceus*, *F. proliferatum* and *Alternaria arborescens* [6,19,24,27,28]. The growth of *B. cinerea* and *A. arborescens*, isolated from tomatoes, was completely inhibited by volatile vaporing at 50 mg L^−1^ for up to 12 h [6]. The *O. vulgare* EO incorporated into the growth medium showed a fungicidal or fungistatic activity at 500 mg L^−1^. Sanit [27] also stated 100% inhibition of crude *O. vulgare* extract on mycelial growth and spore germination of *Alternaria* spp. at all concentrations (1000–10,000 μL L^−1^). However, the most extensive investigations have been completed against pathogenic bacteria [21]. Unfortunately, studies emphasizing the effects of different subspecies of oregano EO on *Alternaria* spp. were not distinguished.

In this study, both EO of OVH and OVV showed moderate ability to control fungal pathogens *Alternaria* spp. However, OVV expressed more potent antifungal activity than OVH EO. Other studies [25] also verified the more potent antimicrobial activity of OVV EO against seven bacterial strains: *Staphylococcus aureus*, *Escherichia coli*, *S. lutea*, *Bacillus cereus*, *Pseudomonas aeroginosa*, *Salmonella typhimurium*, *Enterococcus faecalis* comparing to OVH EO. However, OVH EO demonstrated promising *C. albicans* growth control. Contrary to our results and earlier findings [25], Askun et al. [24] reported that OVH methanol extracts had 100% inhibition against four potential mycotoxigenic fungi: *A. niger*, *A. ochraceus*. *A. flavus*, and *F. proliferatum*. Meanwhile, OVV extract demonstrated fungistatic activity against three fungi (*A. niger*, *A. ochraceus* and *F. proliferatum*). *A. flavus* was resistant to this extract in all concentrations [24]. Additionally, the OVV extract effectively suppressed microbial growth of *S. enteritidis*, *E. coli*, *Listeria monocytogenes*, *S. aureus*, *A. niger* and showed an excellent antifungal effect [22]. Another study [23] noted that OVH collected from different localities had vigorous antimicrobial and antifungal activities against all tested microorganisms. However, the best result was achieved on *Penicillium expansum*, *A. flavus* pathogens, and the growth of *Alternaria brassicicola* was moderately suppressed by this EO.

Little is known about the mechanism of action of oregano EO on *Alternaria* species. Although, there are reported data showing terpenoids’ dose-dependent ability to inhibit mycelial growth, conidial germination, induce cell membrane dysfunction, and interfere with cell metabolism [29]. For example, thymols alone or in combination with carvacrol cause structural and functional disturbances in the cellular membrane. Thymol is a lipophilic compound that can change the cell membrane fluidity and permeability alone or with carvacrol [30]. Lambert et al. [31] highlighted that a mixture of carvacrol and thymol at proper amounts might exert the total inhibition against *Pseudomonas aeruginosa* and *Staphylococcus aureus* that is evident by oregano EO. Such inhibition is due to damage in membrane integrity, which further affects pH homeostasis and the equilibrium of inorganic ions [31].

In our study, the antifungal activity of EO, with main constituents of carvacrol or sabinene, β-caryophyllene, and germacrene D [26], was dose-dependent and manifested by inhibition of *Alternaria* mycelial growth. On the other hand, terpinen-4-ol and germacrene D, β-caryophyllene, and spathulenol were major components in OVV produced in other countries [25]. Due to these differences in the composition of the EO, antifungal effects on pathogens may alter. For example, Abbaszadeh et al. [32] revealed that for the pathogen *Alternaria alternata* 5224 from Persian Type Culture Collection (PTCC), the minimum inhibitory concentrations of synthetic carvacrol 350 μL mL^−1^ or thymol 400 μL mL^−1^ are needed.

This research gives possible directions for further studies evaluating the antifungal mechanism of EO. It is indicated that the advantage of natural products from plants is their multicomponent composition providing a lower possibility for the pathogen to develop resistance quickly. Based on the results in our study, the *O. vulgare* subsp. *vulgare* plants can be considered a potential source of alternative products to the pesticides currently used to prevent fungal decay in fresh products and improve food safety.

## 4. Materials and Methods

### 4.1. Fungal Material

The *Alternaria* spp. isolates used in the experiments were isolated from rotten carrot roots. The carrot root fragments were surface-sterilized for 3 min in 70% ethanol, rinsed five times with sterile distilled water, placed on PDA (potato dextrose agar) (Liofilchem, Teramo, Italy), and incubated at 25 °C. Pathogens obtained from infected carrots were identified using 10× and 40× microscope magnification (Nikon Eclipse 80i, Melville, New York, NY, USA), evaluating their sporangiophores, sporangia, hyphae, conidiophores, conidia, colony texture, and growth pattern [33] and then transferring fungal mycelium onto a new PDA plate for purification to a single-spore. Isolates were incubated at 25 ± 2 °C in the dark for 7 days and kept at 4 °C until the beginning of the experiment.

### 4.2. Essential Oils Extraction

Clevenger-type (Glassco, Ind) hydro-distillation was used for the extraction of EO from local fresh material [26,34,35]. Greek oregano (*O. vulgare* subsp. *hirtum* (Link) Ietsw), and oregano (*O. vulgare* subsp. *vulgare* L.) used for essential oils extraction were grown at Lithuanian Research Centre for Agriculture and Forestry (LAMMC) Institute of Horticulture (IH) experimental fields. The composition of the volatile compounds responsible for antifungal activities of each essential oil was determined previously using gas chromatography/mass spectrometry [26]. The main component of the *O. vulgare* subsp. *hirtum* was carvacrol, *O. vulgare* subsp. *vulgare*—sabinene, β-caryophyllene, and germacrene D [26].

### 4.3. Evaluation of Essential Oils Efficiency

The research was carried out at the Laboratory of Plant Protection LAMMC, IH. The radial growth technique was used to evaluate the EO efficiency on *Alternaria* spp. in vitro [18,36,37]. Appropriate volumes of each EO of OVH and OVV were added to PDA medium immediately before it was poured into the Petri dishes at 40–45 °C to obtain a series of concentrations (200, 400, and 600 µL L^−1^). Four repetitions with four replications were carried out [38]. Under aseptic conditions, 5 mm mycelium plugs (upside down) of single spore 7-day old fungus on PDA plates isolated from carrots were placed in the center of a sterile Petri dish containing PDA and different EO concentrations [18,36,37]. The control treatments were without EO.

**Figure 3:**
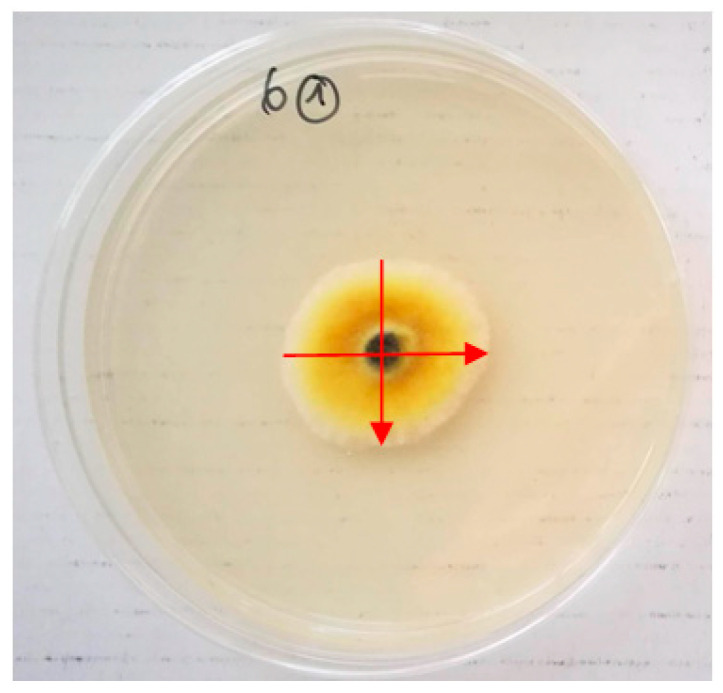
The measurement of the diameter of *Alternaria* spp. mycelium growth.

Plates were incubated at 25 ± 2 °C in the dark, and the diameter of colony growth (cm) was measured in two directions (transverse and longitudinal) after 1, 2, 3, and 7 days of incubation (DAI) (Figure 3).

The mean of colony growth diameters (cm) used for radial colony growth rate (cm/day) calculations [39]:

(1) $$Cr=\frac{r}{t},$$

*Cr*—radial colony growth rate, cm/day; *r*—mean of colony diameters, cm; *t*—growth duration, days.

Radial colony growth rates were used for mycelial growth inhibition calculations using the formula by Šernaitė et al. [40] with some modifications:

(2) $$\mathrm{mycelial}\;\mathrm{growth}\;\mathrm{inhibition}\;\left(\%\right)=\frac{\left(rrc-rrt\right)}{rrc}\times 100,$$

*rrc*—radial colony growth rate of the control (cm/day); *rrt*—radial colony growth rate of the treated samples (cm/day).

### 4.4. Statistical Analysis

All analyses consisted of four repetitions with four replications. Data were statistically handled by one-way analysis of variance (ANOVA) and Duncan’s multiple range test (*p* < 0.05) was used for the comparison of obtained means. Other calculations were completed using Microsoft Excel.

## Figures and Tables

**Table 1:** Mycelial growth inhibition of *Alternaria* spp. by different essential oils compared to the control at 1, 3, and 7 days after inoculation.

Essential Oil	Concentration μL L^−1^	Inhibition of *Alternaria* spp., %		
		1 DAI	3 DAI	7 DAI
*Origanum vulgare* subsp. *vulgare*	200	14.29	1.96	3.17
	400	52.38	3.92	4.76
	600	85.71	11.76	9.52
*Origanum vulgare* subsp. *hirtum*	200	47.06	6.50	0.34
	400	64.71	8.94	0.34
	600	47.06	6.34	1.13
